# New Perspectives of Hydrogels in Chronic Wound Management

**DOI:** 10.3390/molecules30030686

**Published:** 2025-02-04

**Authors:** Adina Alberts, Andreea Gabriela Bratu, Adelina-Gabriela Niculescu, Alexandru Mihai Grumezescu

**Affiliations:** 1 Carol Davila University of Medicine and Pharmacy, 050474 Bucharest, Romania; adina-magdalena.alberts@rez.umfcd.ro; 2 Faculty of Chemical Engineering and Biotechnologies, University Politehnica of Bucharest, Gh. Polizu St. 1-7, 060042 Bucharest, Romania; andreea.bratu2910@stud.fim.upb.ro (A.G.B.); adelina.niculescu@upb.ro (A.-G.N.); 3 Research Institute of the University of Bucharest—ICUB, University of Bucharest, 050657 Bucharest, Romania

**Keywords:** hydrogels, wound healing, chronic wounds, diabetic foot ulcers, stimuli-responsive hydrogels, antibacterial peptides, cytokines

## Abstract

Chronic wounds pose a substantial healthcare concern due to their prevalence and cost burden. This paper presents a detailed overview of chronic wounds and emphasizes the critical need for novel therapeutic solutions. The pathophysiology of wound healing is discussed, including the healing stages and the factors contributing to chronicity. The focus is on diverse types of chronic wounds, such as diabetic foot necrosis, pressure ulcers, and venous leg ulcers, highlighting their etiology, consequences, and the therapeutic issues they provide. Further, modern wound care solutions, particularly hydrogels, are highlighted for tackling the challenges of chronic wound management. Hydrogels are characterized as multipurpose materials that possess vital characteristics like the capacity to retain moisture, biocompatibility, and the incorporation of active drugs. Hydrogels’ effectiveness in therapeutic applications is demonstrated by how they support healing, including preserving ideal moisture levels, promoting cellular migration, and possessing antibacterial properties. Thus, this paper presents hydrogel technology’s latest developments, emphasizing drug-loaded and stimuli-responsive types and underscoring how these advanced formulations greatly improve therapy outcomes by enabling dynamic and focused reactions to the wound environment. Future directions for hydrogel research promote the development of customized hydrogel treatments and the incorporation of digital health tools to improve the treatment of chronic wounds.

## 1. Introduction

Skin wound healing is a complex process that requires the interaction of several highly regulated factors implied in restoring damaged skin and its barrier function. A chronic wound may occur if it has not progressed to heal after 4–12 weeks despite treatment, resulting in considerable strain on the patient and healthcare system [1,2,3]. Chronic wounds have a negative influence on the global community. According to estimations, 8 million people worldwide are suffering from wounds, regardless of whether they are infected. The Global Burden of Disease, which studies over 195 countries and territories, shows that the prevalence of skin and subcutaneous diseases has rapidly increased over a decade, reaching 605,036,000 in 2015 from 492,883,000 in 2005 [4]. Although chronic wounds (30.5%) have a similar 5-year death rate to cancer (31%), funding for research into these life-threatening consequences is substantially lower than for cancer research [5]. Patients can experience intense pain, mental and physical discomfort, limited movement, and social isolation during this period. Chronic wounds can lead to disability, necessitating amputation after all effective treatments have been exhausted. Diabetic ulcers cause amputations every 30 s worldwide [6]. Socioeconomic position (e.g., salary, education, and employment status), physical environment, psychosocial aspects (stress, depression, etc.), and social support networks all interact with an individual’s biology to determine health outcomes such as disease development, wound healing, and life expectancy. Food insecurity, defined as a lack of continuous access to enough food to support a healthy lifestyle, is a major issue affecting 11% of U.S. individuals and is linked to chronic illnesses including obesity; diabetes; and, by extension, wound healing [7,8].

The incidence of chronic wounds rises with age, especially for people with diabetes and obesity, who are more likely to develop one due to several factors, such as neuropathy, autoimmune and cardiovascular diseases, and hyperglycemia. The U.S. government forecasts that the senior population will exceed 77 million by 2060, implying that chronic wounds will remain an increasingly prevalent concern in this demographic [9,10].

Significant economic impacts are linked to chronic wounds, including direct expenses to society (medical and healthcare expenses) and indirect expenses (diminished productivity due to early retirement and sick leave). The global advanced wound care market is expected to reach USD 30.52 billion by 2030, with an annual growth rate (CAGR) of 4.61% from 2023 to 2030 [8]. The costs associated with treating chronic wounds are high and are estimated to be between 1% and 3% of all healthcare spending in affluent nations [3,9,10,11]. Numerous modern technologies have been explored due to the demand for more intricate biology of chronic wounds and biomimetic techniques. However, an in-depth understanding of the wound healing process and its main characteristics is necessary to develop novel therapeutics. The presence and function of various stem cells, in addition to other engaged mediators, including biopolymeric porous scaffolds, films, hydrogels, and electrospun nanofiber meshes, are combined with the conventional treatment to provide an upgraded description of the wound healing process [2,12].

Skin represents the human body’s largest single organ, with a surface area of around 2 m^2^ and a mass equivalent to approximately 15% of total body mass. It is involved in various processes, including defense against pathogens and chemicals, hydration, excretion, synthesis of vitamin D, and thermal regulation. The process through which a skin wound heals demonstrates a remarkable cellular function mechanism that can be separated into four stages: hemostasis, inflammation, proliferation, and remodeling [13,14].

Immediately after wounding, clotting cascades are started, and vasoconstriction is initiated for five to ten minutes, preventing the wound from bleeding. Additionally, fibrin clog generates a temporary matrix that functions as a scaffold structure supporting subsequent healing processes, including the migration of endothelial cells, fibroblasts, keratinocytes, and leukocytes, as well as growth factors. Following this brief vasoconstriction response, vasodilatation occurs, resulting in localized hyperemia and edema [15,16,17]. The inflammatory phase begins a few hours after injury and is triggered by bacterial products, released cytokines and chemokines, and mediators produced from platelets. An increase in vascular permeability causes neutrophils to enter the wound site first, releasing proteases such as matrix metalloproteinases (MMPs), reactive oxygen species (ROS), antimicrobial peptides, and growth factors (TGF-β) to eliminate bacteria and remove damaged protein matrices. To intensify the inflammatory process, monocytes come afterward (within 24 h) and mostly change into pro-inflammatory M1 macrophages [2,18]. Lymphocytes are recruited later, with γδ + T cells supporting the proliferation and viability of fibroblasts, immune cells, and keratinocytes, including insulin growth factor 1 (IGF-1), keratinocyte growth factors (KGFs), and fibroblast growth factors (FGFs). αβ + T cells (CD4+, CD8+, and Treg) are crucial in combating harmful microbes. In the final stages, M1 macrophages transition to anti-inflammatory M2 macrophages, releasing IGF-1, TGF-β1, VEGF, PDGF, and IL-10 factors to promote cell migration, proliferation, and matrix formation [19,20].

The proliferative phase begins when inflammation decreases, with monocytes and macrophages transforming into anti-inflammatory cell types (M2 macrophages) primarily due to immunomodulatory mediators released by mesenchymal stem cells (MSCs). Granulation tissue growth is the main objective of this phase, as it covers the deprived wound surface and helps to restore the vascular network. At this stage, fibroblasts are controlled by growth factors and cytokines, including TNFα, TGFα/β, PDGF, FGFs, EGF, connective tissue growth factor (CTGF), and IL-1. They deposit a lot of extracellular matrix (ECM) to fill in tissue gaps while also releasing more cytokines such as hepatocyte growth factor (HGF), FGFs, IFNs, TGFβ, and VEGF [21,22]. Vasculogenesis and angiogenesis contribute to the neovascularization process reaching its peak in the current phase, resulting in premature granulation tissue with high fibroblasts, M2 macrophages, and irregularly arranged collagen bundles [23]. Remodeling represents the last stage of the healing process that endures for a year or longer and is distinguished by a progressive loss of cellularity and vasculature. Fibroblasts increase the level of type I collagen and the ECM of various components, while MMPs are responsible for breaking down the disorganized type III collagen, forming scars [2,24].

**Figure 1:**
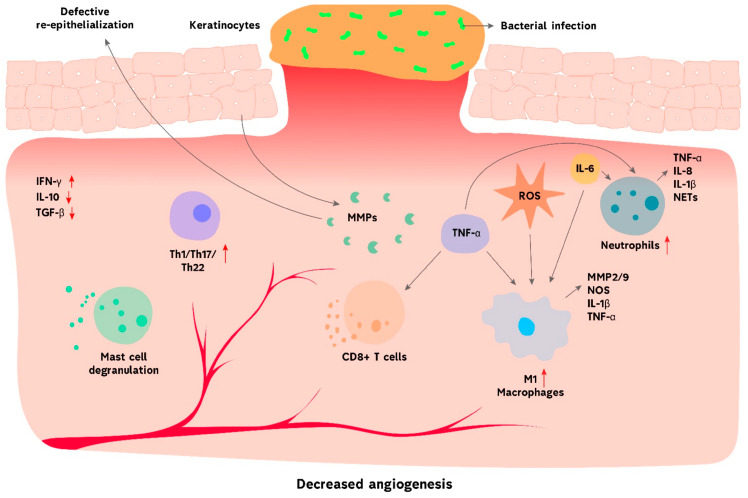
Immune system in the healing of chronic wounds. Reprinted from an open-access source [31].

However, the healing of skin tissue is delayed when wounds fail to comply with this structured pattern, ultimately leading to chronic wounds and demanding a coordinated sequence of immunological activities. Typical characteristics of non-healing wounds are represented by exudation, recurrent infection, tissue necrosis, impaired re-epithelialization, reduced angiogenesis, and excessive ROS generation [25,26]. The extended occurrence of myeloid cell populations, including neutrophils, monocytes, and macrophages, throughout the late stages of inflammation indicates chronic wound healing (Figure 1). On the other hand, as the process advances, the proportion of eosinophils, dermal dendritic cells, and Langerhans cells (LCs) decreases, while mast and T cells contribute to the pro-inflammatory nature of chronic wounds. Chronic inflammation is characterized by increased expression of the CXCR3 ligand, which is present in Th1 cells [27,28]. By releasing several signaling molecules, immune cells actively interact with non-hematopoietic cells, including keratinocytes. Although the exact mechanism is unclear, keratinocytes play a crucial role in developing chronic wounds. Defective regulation of miRNAs in keratinocytes has been shown to affect immune responses and cause cutaneous wounds to heal slower [29]. The miRNAs epigenetically control chronic wound development by modulating pathways such as NF-κB, PI3K/Akt/mTOR, TGF-β/Smad, VEGF, and Wnt/β-catenin [30].

Wound dressings are essential in the wound recovery process because they cover the wound’s area against the external environment. They can also interact with the wound bed to promote and accelerate the healing process. Innovative dressings such as hydrogels are developed to encourage wound healing by establishing the ideal microclimate between the dressing and the wound bed through their moisture-exchanging properties. These dressings also have a cooling, calming effect and lessen the discomfort of changing dressings because of their high moisture content. Hydrogel dressings’ transparency enables clinical assessment of healing without removing the dressing. Furthermore, hydrogels can be easily wiped away from the wound without further damaging the healing tissue [32,33]. Because of these unique characteristics, hydrogel dressings can be used on numerous chronic wounds.

In this context, it is essential to address the complexity of chronic wounds, including moisture retention and balance, autolytic debridement, infection control, and sustained drug delivery directly to the wound site. This review concentrates on innovative strategies in smart hydrogels with advanced drug delivery systems or antimicrobial properties that can improve the customization of the treatments based on the wound type and its stage. These developments can help researchers stay up to date with state-of-the-art technologies and promote further innovations such as 3D printing and AI.

## 2. Chronic Wound Types

Chronic wounds can generally be divided into three major categories: diabetic foot ulcers (DFU), vascular ulcers, and pressure ulcers. They tend to occur in older adults with pathological diseases such as obesity, vascular disease, and diabetes mellitus [31].

The International Working Group on Diabetic Foot (IWGDF) defined DFU as a collection of symptoms secondary to either current or past diabetes, such as skin dryness, ulceration, infection, or destruction of foot tissue [34,35]. Numerous diabetic patients suffer from DFU, a complex and multifactorial clinical issue that causes ulceration and infection, usually accompanied by neuropathy and/or peripheral artery disease (PAD). The normal functioning of the foot is directly impacted by motor neuropathy, which denervates certain muscle groups, causing atrophy of the foot muscles. PAD contributes significantly to neuropathy, leading to leg ulcers and amputations. Because diabetes mellitus (DM) primarily affects the arteries of the lower limbs, the frequency of lower limb amputations is higher in patients with PAD than in those without PAD. After the ulcer has developed, the conditions influencing its healing may become more complicated, with various factors taking precedence at different times. These ulcerations and infections damage the foot’s epidermis and dermis, rupture the skin’s protective layer, reveal sterile structures, and ultimately develop full-thickness lesions. The score and classification criteria for characterizing DFU lesions were structured in a widely accepted method in clinical practice [35,36]. One of the recent systems is represented by The Wound, Ischemia, and foot Infection system. The three most significant risk factors that could result in lower limb amputation are covered by this system. Each of the three factors is given a score between 0 and 3, with the wound being graded according to its size, depth, severity, and expected difficulty in healing; ischemia being rated according to the ABI grading; and foot infection being rated according to its depth and scope. Based on clinical research, this approach is most helpful in forecasting severe amputations [37].

Pressure ulcers (PUs), also referred to as bedsores, decubitus ulcers, pressure sores, or pressure injuries, are lesions caused by localized disruption of circulation. PUs can result from prolonged pressure on a body component caused by a shearing force, from the body’s weight or a limb, or both, particularly in nutritionally deficient patients. Long-term immobilization on a bed, table, or spinal board and improperly fitting medical equipment encountering patient tissues are examples of extrinsic risk factors. Intrinsic risk factors like smoking, diabetes, and malnutrition further raise the general danger of pressure ulcers. PUs can cause swelling of the skin (category I, non-blanchable erythema), broken skin (category II, partial thickness skin loss), and deep ulcers with exposed bone, tendon, or muscle (category III, full-thickness skin loss, or category IV, full-thickness tissue loss) [38,39].

To effectively treat PUs, multidisciplinary teams should unite specialists in plastic surgery, physical therapy, infectious diseases, orthopedics, internal medicine, general surgery, and neurology. Nowadays, the primary surgical treatment method is radical debridement, followed by flap closure with sufficient volume and a sufficient blood supply. This procedure is becoming less popular because skin grafting increases the risk of ulcer recurrence. Recurrence rates might occasionally surpass 80% despite advancements in surgical therapies, suggesting that this significant issue has not yet been completely addressed [40].

Venous leg ulcers represent the most severe form of chronic venous illness induced by venous hypertension. Venous ulceration is currently controlled by a mix of microscopic and macroscopic pathologic processes. Macroscopic changes are pathologic processes associated with cellular abnormalities, varicose vein development, and vein wall architecture that compromise venous function. These processes, which result in venous hypertension and the breakdown of normal vein wall architecture, are mostly brought on by hereditary causes. Chronic inflammation caused by venous hypertension can eventually result in venous ulcers. Chronic extravasation of macromolecules, products of red blood cell breakdown, and iron excess are the triggers for inflammatory damage. White blood cell migration into the dermis and the release of several pro-inflammatory cytokines are caused by chronic inflammation. These cytokines change the fibroblasts’ phenotype to one that is more contractile, which raises dermal tension. Furthermore, iron overload maintains the M1 phenotype of macrophages, which results in tissue damage instead of dermal healing. The main goals of current medicinal and surgical treatments are to eradicate venous hypertension and encourage the healing of venous ulcer wounds. Despite advancements in understanding venous ulcer genesis and repair, ulcers continue to typically require 6 months to cure and have a recurrence rate of more than 58% after 5 years [41,42].

Among other chronic wounds, arterial leg ulcers are also mentioned. They account for approximately 22% of all ulcers and are caused by inadequate blood supply to the legs due to blockages or constriction of arteries (atherosclerosis). In the absence of treatment for the underlying inadequate arterial blood supply, ulcers may never heal or may heal slowly [43]. Moreover, vascular mixed ulcers account for around 10% of lower leg ulcers. However, their incidence is underestimated. The therapy of venous and arterial abnormalities represents a combination of physiopathological and anatomical aspects. To ensure optimal treatment, both need to be carefully examined [44].

## 3. Challenges in Chronic Wound Management

The treatment for the aforementioned chronic wounds is ineffective when a persistent infection occurs, despite rigorous care. Numerous aerobic and anaerobic pathogenic microorganisms, such as *Pseudomonas aeruginosa*, *Staphylococcus aureus*, *Candida albicans*, and β-hemolytic streptococci, are involved in the microbial colonization of the wounds due to impaired host immune response resulting in delaying the wound healing. Additional evidence supporting the causal function of biofilms in chronic wounds has come from investigations of the relationship between bacteria and wound tissue in keratinocyte cells in vitro and in animal wound models in vivo. It is crucial to investigate biofilms in chronic wounds using animal models, which are especially useful for addressing concerns of correlation and causation regarding wound healing in the presence of biofilms. To prevent infection, wounds require treatment with aseptic procedures, adequate debridement, and suitable antimicrobial agents. Topical antimicrobials are becoming critical in wound care due to the increase in antimicrobial resistance brought on by antimicrobial abuse, particularly systemic antibiotic use. Due to their direct application to the wound, topical antimicrobials have a high concentration at the site of the lesion, few systemic adverse effects, and a low rate of antimicrobial resistance [45,46,47,48].

The complex wound-healing procedure can be affected by various conditions, including bacterial infections, illnesses, hypoxia, and chronic inflammation. Chronic wounds are characterized by chronic inflammation linked to immunological modulation. The lack of immune system regulation results in ongoing inflammation and decreased wound healing, leading to chronic skin wounds. Hypoxia, a lack of oxygen supply to the wound bed, represents another important factor in wound healing. Acute hypoxia stimulates angiogenesis by increasing the synthesis of hypoxia-inducible factor-1 (HIF-1) and its target gene, vascular endothelial growth factor (VEGF). In contrast, chronic hypoxia not only impairs angiogenesis but also reduces cell signaling, which can contribute to delayed wound healing. Various reasons, including insufficient blood flow, tissue injury, and inflammation, can cause hypoxia. By preventing several phases of healing, such as angiogenesis, cell proliferation, and re-epithelialization, hypoxia can impede tissue regeneration, postpone wound healing, and raise the risk of infections. Therefore, there is an urgent need for efficient methods to combat hypoxia and promote wound healing [49,50].

## 4. Hydrogels: An Overview

**Figure 2:**
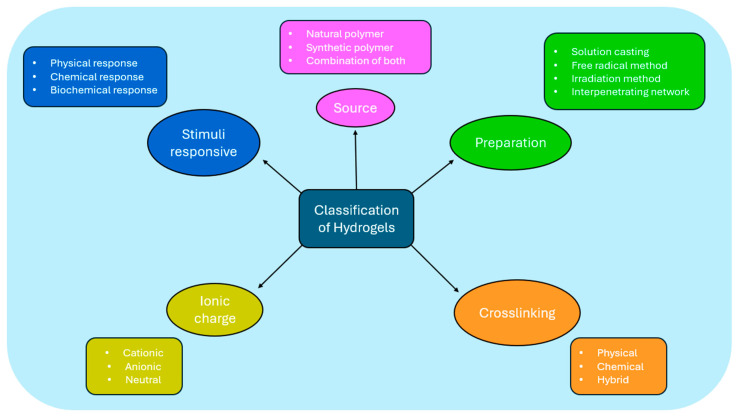
Classification of hydrogels. Created based on information from open-access sources [53,54,55].

Hydrogels are three-dimensional, crosslinked, hydrophilic polymer structures that absorb, swell, and hold huge volumes of water or aqueous fluids. The hydrophilicity and degree of crosslinking of the polymer chains are two crucial factors that affect the properties of hydrogels. Functional groups, including hydroxylic (-OH), carboxylic (-COOH), amidic (-CONH–), primary amidic (-CONH_2_), and sulphonic (-SO_3_H) groups present in the polymer network provide hydrogels their capacity to hold water. Hydrogels can be synthesized by copolymerizing and crosslinking one or more functional monomers. Crosslinking can be a chemical, physical, or simultaneous process and may be produced by various methods, including interpenetrating network generation, bulk polymerization, free radical polymerization, UV and gamma irradiation, solution casting, and simple mixing. The primary synthetic ingredients of hydrogels are typically an initiator, monomer, and crosslinker. By altering the synthetic parameters, such as the initiator concentration, monomer concentration, reaction temperature, reaction vessel, reaction time, and crosslinker to monomer ratio, hydrogel properties can be controlled [51,52,53]. Hydrogels can be categorized according to several characteristics, as presented in Figure 2.

Hydrogels are classified based on the materials (polymers) employed, the polymer source, the crosslinking process, the responsiveness to stimuli, and the ionic charge. The hydrogels contain natural, synthetic, or a mix of both polymers. These polymers can produce a variety of hydrogels, including terpolymers, homopolymers, copolymers, block copolymers, and interpenetrating network (IPN) hydrogels. Homopolymer hydrogels are composed of a single type of hydrophilic monomer unit, while copolymer hydrogels are made up of two comonomer units, one of which must be hydrophilic for water swelling. The reaction of three or more comonomers results in multipolymer hydrogels. Lastly, there are two ways to create interpenetrating network (IPN) hydrogels: in solution and within a premade network. The most popular technique involves polymerizing a single monomer within a distinct crosslinked hydrogel network. After the monomer polymerizes, a polymer or another crosslinked network is created and then interwoven with the original network [56,57]. A comparison between the various types of hydrogels, including their cost, biocompatibility, efficiency, advantages, and limitations, is presented in Table 1.

Natural hydrogels consist of natural polymers such as polysaccharides (alginate, chitosan) and proteins (gelatin, collagen, lysozyme) [87]. Natural polymers are ideal for biomedical applications because they are highly biocompatible, biodegradable with high cell adhesion capabilities, and contain biologically identified moieties. Chitosan and cellulose are two representative examples of this class of polymers that possess antibacterial properties and offer promising research opportunities as a typical wound dressing material. However, these natural polymers’ antibacterial properties are insufficient for therapeutic use. Thus, a large number of hydrogels were loaded with antibacterial and antibiotic agents (such as metal nanoparticles, common medications, etc.), and cutting-edge technologies were employed to create a range of innovative multifunctional hydrogels with strong antibacterial and irritant drug release [88].

Despite the advantages presented, natural polymers lack the required mechanical characteristics, which must be similar to the application location. Additionally, when natural polymers are introduced into the human body, they may cause immunological and inflammatory reactions. Synthetic hydrogels, including poly (hydroxyethyl methacrylate) (PHEMA), polyethylene glycol (PEG) hydrogels, and polyacrylic acid (PAA), are produced by polymerizing several kinds of synthetic monomers. However, synthetic polymer hydrogels can be engineered to produce the specified mechanical characteristics and other desirable properties, even though they are deficient in inherent bioactivity [56,66]. Polyvinyl alcohol outperforms natural polymer hydrogels in terms of mechanical characteristics. As a wound dressing, PVA hydrogels may protect the wound and prevent second injuries caused by external environmental stimuli and mechanical force applied to the wound, making them suitable for clinical use. Additionally, PVA hydrogels have a high moisture content and strong permeability to oxygen and water, which may serve to keep a wound wet as it heals and encourage the formation of new tissue. Nevertheless, the antibacterial effectiveness of PVA hydrogels as dressing materials must be improved, as the hydrogels themselves possess antibacterial qualities. Antibacterial modification primarily involves inorganic, organic, and natural antibacterial compounds. PVA hydrogel systems’ antibacterial components are often pricy, and their modification techniques are challenging. Thus, the researchers focus on creating antibacterial PVA hydrogels using a low-cost, straightforward, and large-scale manufacturing approach [89,90,91].

Hybrid hydrogels involve functionally, morphologically, and chemically distinct structural elements, such as biologically active peptides, proteins, or micro/nanostructures, linked together through chemical or physical mechanisms. Hybrid hydrogels that are compatible in vitro (for studies of cell proliferation, differentiation, and migration) and in vivo (for wound healing, tissue engineering, and drug delivery) are generally produced by conjugation or polymerization techniques between peptides and proteins incorporated into networks [92]. Hydrogels respond to various stimuli, allowing for dynamic modification of their characteristics and behavior in various applications. The formulations are responsive to one or more stimuli, including pH, ions, redox, light, potential, temperature, electric field, and magnetic field [93]. According to their ionic charge, hydrogels can also be categorized as neutral (uncharged), cationic (only carrying positive charges), anionic (only carrying negative charges), and ampholytic, meaning they have both positive and negative charges. The polymer’s charge determines the network’s overall charge [53,57]. Hydrogels can also be categorized as either (1) amorphous hydrogels, which have covalent crosslinks, or (2) semicrystalline hydrogels, which can or cannot possess covalent crosslinks, depending on the network’s physicochemical structural characteristics. The macromolecular chains in amorphous hydrogels are organized randomly. Regions of organized macromolecular chains (crystallites) that self-assemble are characteristic of semicrystalline hydrogels [57].

Hydrogels’ diverse biological, physicochemical, and structural properties have led to their current exploration and use in various biomedical applications. One of the most well-known industries is aesthetic medicine, where many commercial hydrogel products—such as hydrogel based on hyaluronic acid—have been used as fillers. Hydrogels have also been widely employed as 3D models of various diseases (such as tumors, tissue fibrosis, corneal disorders, nerve diseases, inflammatory bowel diseases, etc.) for high-throughput drug screening or pathogenesis studies. Hydrogels are advantageous for encapsulating cells and expansion both in vitro and in vivo because of their ability to imitate the stroma matrix of in vivo tissue. This allows for highly effective tissue regeneration and cancer treatment. Hydrogels are thought to be appropriate drug carriers for controlled and sustained release at locations of interest, as well as therapy efficiency evaluation. Additionally, when hydrogels combine with functional units, they can associate with conductive wearable/implantable biodevices, biosensors, and bioimaging. The efficacy of hydrogel-based therapy has also been validated by several clinical trials in many conditions, including severe heart failure, type 2 diabetes, chronic renal disease, oral-maxillofacial and orthopedic trauma procedures, knee osteoarthritis, and spinal fusion [94,95].

## 5. Mechanisms of Action in Wound Management

Functional hydrogels have gained significant attention in wound dressing research. These hydrogels can display high-performance biological activities, such as bioadhesivity, antibacterial properties, blood coagulation, and regeneration promotion, which are essential for maintaining stability over time. One of the primary characteristics of hydrogel dressings is their moisture content, which affects how quickly exudates are absorbed and how wet the dressing keeps the surrounding area. This characteristic keeps the wound’s moisture content at ideal levels and improves hemostasis. Hydrogels can act as a partial template during the re-epithelialization and remodeling of chronic wounds because of their biodegradability and biocompatibility. Additionally, hydrogels offer a flexible framework for adding different substances like medications, antibiotic and antibacterial agents, and other extra biomolecules, increasing their overall effectiveness in accelerating wound healing. Therefore, it can be considered that materials based on hydrogel have the maximum degree of suitability for use as dressings to cover wounds in the skin [94,96,97].

Hydrogels are extremely cytocompatible and can be easily modified to have the necessary degradability, stable viscoelasticity, and cell adhesion ligands. Rigidity is a key factor in cell viability, with higher rigidity resulting in faster cell proliferation in cell cultures. Furthermore, cell differentiation is influenced by the stiffness of the microenvironment, which affects gene expression through cytoskeleton protein adhesion and fate tuning [60]. Microbial infections represent the main factors preventing wounds from healing, especially chronic ones that require difficult treatment. Hydrogels can provide antimicrobial action, avoiding or slowing down microbial infections. Since natural hydrogels effectively suppress bacterial infections, they are regarded as very useful biomaterials. Antimicrobial factors (antibiotics, nanoparticles, extracts, etc.) can be loaded into hydrogels’ porous structure, which can be adjusted by varying the crosslinking density. This allows for adjusting the antimicrobial agent’s release rate, which is influenced by the factor’s diffusion coefficient through the gel network [64]. Hydrogels can protect immune cells from death by releasing cytokines, and they can eventually attract the host immune cells and trigger an enhanced immunological response. They hold promise for immunotherapy against autoimmune disorders and infections. Furthermore, hydrogels with interconnected pores can effectively boost immune cell responses and regulate the release of cytokines, growth factors, and adjuvants. By blocking immune cell maturation, activity, and/or apoptosis, the capacity to release these bioactive chemicals or cells locally suppresses immunological responses and promotes immune tolerance [98].

**Figure 3:**
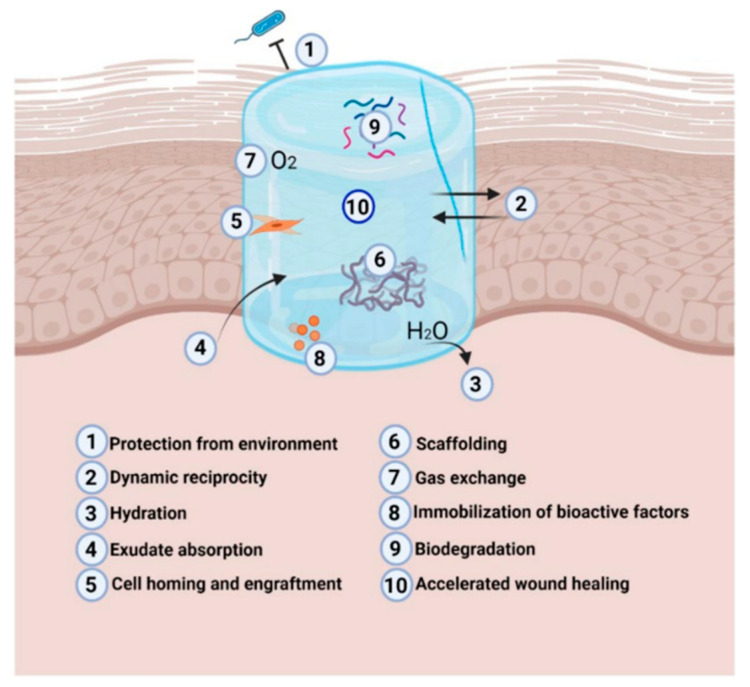
Hydrogel dressings’ therapeutic impact on wound healing. Reprinted from an open-access source [99].

The extraordinary capacity of hydrogels to keep oxygen, absorb wound exudate, and preserve the moist environment that promotes normal physiological activities in the wound bed makes them unique in producing successful DFU dressings. Their porous nature facilitates sufficient gas exchange, enabling the wound to breathe during the healing and closure processes (Figure 3) [99].

## 6. Applications of Hydrogels in Chronic Wounds

The therapeutic impact of hydrogels obtained from natural polymers such as collagen, alginate, or chitosan in the context of diabetic wound healing has been extensively studied in recent years. In diabetic rat models, for example, Lei et al. [100] assessed collagen hydrogels’ capacity to stimulate angiogenesis and wound healing. This was accomplished by creating full-thickness wounds and treating them externally with a collagen hydrogel that contained recombinant human epidermal growth factors. Following a 14-day treatment period, the rats administered with the artificial hydrogel displayed noticeably lower wound areas than the group that was not given hydrogel treatment, suggesting faster damage regeneration. Regarding angiogenesis, the suggested hydrogel dressing promoted the production of vascularized scar tissue and endogenous collagen synthesis. Alginate hydrogels were designed by Tellechea et al. [101] to encapsulate human umbilical cord-derived outgrowth endothelial cells (OECs) and two biological compounds with anti-inflammatory properties: substance P and neurotensin. Testing in a mouse model with diabetes showed that the alginate hydrogels allowed for sustained neuropeptide release for the 10-day research, which led to an impressive 80% reduction in wound size. A 40% reduction in wound size after 4 days of therapy indicated a synergistic effect promoting neovascularization, demonstrating the additional increased and faster wound healing caused by integrating vascular endothelial growth factor (VEGF) into the recommended hydrogel. A significant promise exists for diabetic wound healing when appropriate immune responses are triggered to promote fast angiogenesis. To take advantage of this, Kai et al. [102] developed an injectable hydrogel containing exosomes that can restore itself using modified chitosan. This exosome-loaded hydrogel appears to have accelerated diabetic full-thickness wound healing by ongoing exosome release, improving angiogenesis and diabetic wound healing and promoting wound healing efficiency. It is characterized by fast re-epithelization, fast collagen deposition, and abundant angiogenesis at the wound sites, suggesting a potential treatment strategy for DFU.

**Figure 4:**
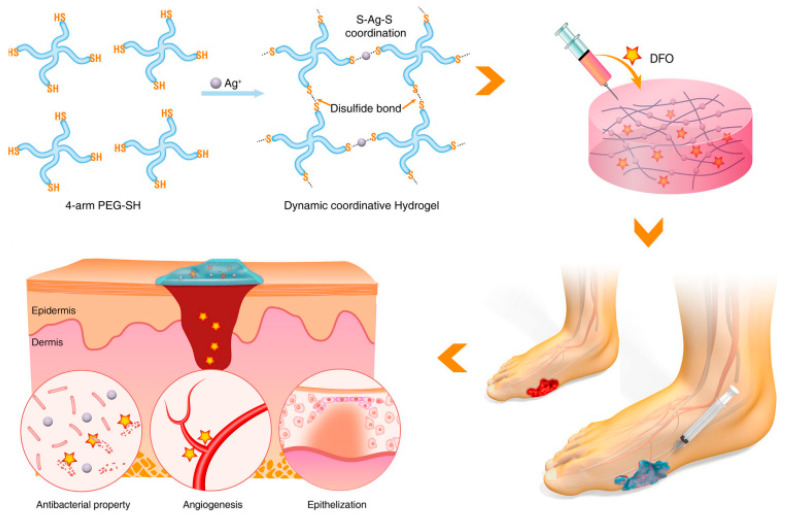
The mechanism and therapeutic effect after the hydrogel injection on type I diabetic foot ulcer. Reprinted from an open-access source [103].

Even though natural hydrogels have exceptional bioactivity, they require significant mechanical performance and reliability improvement. In this regard, synthetic polymers are more adaptable compounds with specific characteristics that carefully regulated chemical or physical procedures may modify. Synthetic polymers are easier to manufacture on an industrial scale than their natural counterparts, and their tunable properties make them ideal for synthesizing various mixtures that promote the desired tissue growth. Furthermore, synthetic polymers can display more uniform architectures and higher water absorption capacities due to precisely controlling their hydrophilic and hydrophobic areas [99]. A multipurpose hydrogel scaffold with injectable, self-healing, antimicrobial, and angiogenic properties for diabetic wound regeneration was described by Chen et al. Multi-arm thiolated polyethylene glycol (SH-PEG) was coordinatively crosslinked with silver nitrate (AgNO_3_) to generate the multifunctional hydrogel. The angiogenic agent desferrioxamine (DFO) was loaded during the cross-linking process. In vitro tests verified the dynamic coordinative hydrogel’s multifunctionality, which included angiogenic potential, resistance to mechanical irritation, high flexibility in manipulation, and antimicrobial characteristics. Furthermore, in vivo research showed that the hydrogel may be injected to effectively heal diabetic skin lesions with increased angiogenic activity and a reduced risk of bacterial infection. The mechanism of the hydrogel in repairing foot ulcers of type I diabetes is illustrated in Figure 4 [103].

To promote skin regeneration in diabetic wounds in mice, Xu et al. [104] developed an in situ polymerizable hydrogel that encapsulated adipose-derived stem cells. The reversible addition–fragmentation chain-transfer (RAFT) polymerization process was used to create hyperbranched multi-acrylated PEG macromers. These macromers were then combined with thiolated hyaluronic acid to create a hydrogel by a thiol–ene click reaction. The therapeutic effectiveness of the proposed system was proved by in vivo testing results that indicated reduced inflammation, higher healing rates, and granular tissue growth.

PVA is a hydrophilic polymer that has gained attention in the biomedical field due to its semicrystalline, biocompatible, and biodegradable properties. Additionally, polyphenols derived from green tea (TPs) have been studied recently using PVA-based hydrogels because of their stability and constant release of natural biomolecules with antioxidant activity. In their research, Chen et al. [105] explored the development of a PVA–alginate composite system that enclosed TPs nanoparticles using a combination of hydrogen bonding and ionic crosslinking. The therapeutic effect of the recommended dressing was assessed on diabetic rats’ excisional linear wounds for seven days. According to the researchers, the hydrogel system’s design encouraged increased collagen accumulation and maturation, and the generation of granulation tissue compared to the control ones.

Pressure ulcers are a particularly difficult problem to address in chronic wounds. The primary goals of treatment are usually to avoid infection while enabling the wound to recover. For this purpose, T. Khampieng et al. [106] developed hydrogel pads using silver nanoparticles. The gel fraction, mechanical characteristics, and swelling capacity of the suggested material must be appropriate for it to be used as a wound dressing; PVP/alginate/chitosan was found to exhibit these qualities. Additionally, when tested against human keratinocytes (HaCaT), L929 murine fibroblasts, and adult human dermal fibroblasts (HDFa), the hydrogel based on silver nanoparticles was found to have antibacterial activity and to be lacking in cytotoxicity. The proposed material was then compared to commercially available dressings, with results indicating that the proposed hydrogel outperformed the commercial alternatives regarding non-cytotoxicity, reduced cost, and swelling, while its capacity to reduce bacteria was equal. As a result, it can be stated that the silver nanoparticle-based hydrogel has significant promise for treating pressure ulcers when used as a wound dressing.

Furthermore, hydrogels with piezoelectric characteristics offer notable benefits because of their exceptional mechanical-to-electric response, blood flow regulation, and angiogenesis stimulation. Ning and coworkers produced a piezoelectric hydrogel for pressure injuries (PIs) or PUs based on Polyacrylonitrile-acrylamide-styrene sulfate-poly (vinylidene fluoride) (PAAN-PVDF). The piezoelectric gels exhibited remarkable mechanical capabilities, skin-like ductility, and exceptional stretchability. Their Young’s modulus values (0.48 0.03) were comparable to human skin’s (0.5–1.95 MPa). Furthermore, the mechanical–electrical properties study showed that the piezoelectric output characteristics were caused by the alignment of the dipole moment between Polyvinylidene fluoride (PVDF) and acrylonitrile at a specific applied pressure. Therefore, these results show that electrical stimulation of the 15% PAAN-PVDF composite PiezoGel increased in vitro angiogenesis, suggesting potential uses for early therapy of PIs or PUs [107].

For better clarity, the above-described studies and additional research initiatives are gathered in Table 2, offering an at-glance perspective on the topic.

## 7. Innovations in Hydrogel Technology

**Figure 5:**
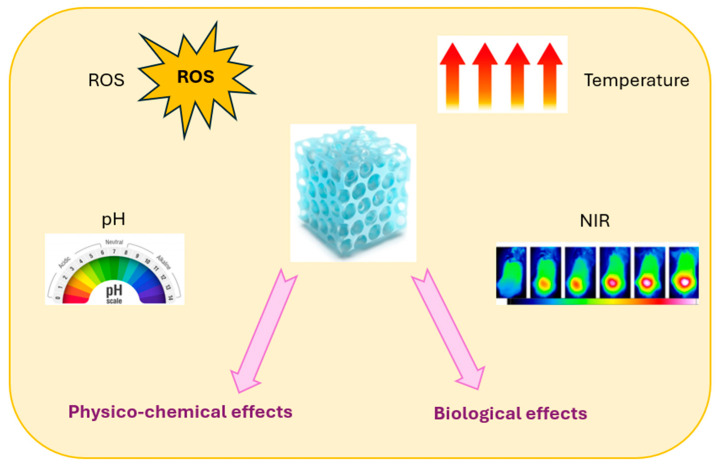
Various factors cause the hydrogels’ reaction. Created based on information from open-access sources [113,114,115].

Innovative hydrogel scaffolds are being developed to tune biomaterial characteristics and achieve real ECM mimics dynamically. One of the most significant subclasses of hydrogels, particularly for development in biological and robotic applications, are stimuli-responsive hydrogels, which alter structurally or mechanically in response to environmental inputs or triggers. There are three types of stimuli-responsive hydrogels: non-contact stimuli-responsive hydrogels (such as those that respond to light, heat, or magnetic or electric fields), contact stimuli-responsive hydrogels (such as those that respond to pH, ions, or chemicals or biochemicals), and multi stimuli-responsive hydrogels (which are susceptible to the simultaneous or sequential action of two or more stimuli). As shown schematically in Figure 5, the most effective and promising stimuli for regulating the behaviors of the resultant hydrogels are pH, temperature, reactive oxygen species (ROS), and NIR. This suggests a variety of uses in drug delivery, separation, sensing, bionic devices, regenerative medicine, and more [111,112,113].

Stimuli-responsive hydrogels are one of the most significant subclasses of hydrogel, particularly for development in biological and robotic applications, since they undergo structural or mechanical changes in response to environmental stimuli or triggers. Recently, research has focused on using microrobots to cure diseases. One of the most important features of developing and constructing microrobots is their ability to move autonomously on a small scale. Therapeutic effectiveness and drug delivery efficiency may be improved by the active movement of medication-loaded microrobots. Microrobot-based active delivery has promising in vivo properties, such as delivering therapeutic payloads to target areas and entering tissue for improved retention, in contrast to conventional passive drug delivery methods, showing immense potential in wound healing and regenerative medicine [116,117].

The aforementioned stimuli may be employed in causing phase transition or stiffness change in hydrogels, which implies a variety of uses other than wound dressing, such as drug administration, sensing, bionic devices, and regenerative medicine [111,118]. Because of their ability to express distinct characteristics, smart/stimuli-responsive hydrogels are frequently used in tissue engineering applications. They act as platforms to facilitate cell migration to the site of damage, have a remarkable ability to replicate the surrounding ECM environment, and may successfully modify their mechano-physical characteristics to meet the application’s needs and their tremendous potential as scaffolds for healing tissue abnormalities. Several bodily tissues, including cardiac, neural, skin, corneal, bone, cartilage, tendon, meniscus, and intervertebral disc, have been rebuilt using stimuli-responsive hydrogels [119,120,121,122,123]. Stimulus-responsive hydrogels are used to provide on-demand release, guaranteeing that growth factors or cells reach certain damaged regions and offer biological and mechanical support for abnormalities in bone and cartilage. Multi-stimuli-responsive hydrogels can be used to distribute bone morphogenetic proteins (BMPs) and other osteogenic factors to specific areas. They can greatly improve bone growth and repair by responding to external stimuli with local signals [124,125]. Smart devices for medication administration and cell transplantation have been designed in cardiac pathology using a variety of disease-relevant environmental cues to aid in heart repair. By simulating the mechanical characteristics of cardiac tissue, these hydrogels can enhance cell activity. Additionally, they can administer therapeutic drugs that encourage angiogenesis or target cardiac damage. Bulk hydrogels, supramolecular hydrogels, and nanoparticles can all be programmed to respond to the same stimulus but have different drug cargos and release mechanisms [126,127]. Multi-stimuli-responsive hydrogels can be programmed to release neurotrophic substances in response to injury-related stimuli. In response to the inflammatory and oxidative stress environment at the injury site, stimuli-responsive hydrogels can control oxidative stress, inflammation, and edema levels while fostering neurogenesis and functional recovery [128].

Different polymeric materials used in hydrogels are engineered to react to particular biological signals. These nanogels are multifunctional and multi-responsive, demonstrating dynamic loading and release of therapeutic drugs, interacting with biological substrates, converting external signals into therapeutic heating, and encouraging cellular internalization. A novel and modular sequence of nanogel modifications containing small molecules, peptides, or proteins was created. In wound healing and regenerative medicine, these materials can be designed for controlled release and targeted medication administration, increasing therapy efficacy while reducing adverse effects. New possibilities for patient-specific treatments are made possible by developing materials that intelligently interact with the biological environment [129,130]. Responsive wound care solutions may be developed by exploiting the physical features of materials, such as a low critical solution temperature, and deliberately matching them with the pathological circumstances commonly encountered in wound settings. Drugs, for instance, can be uniformly distributed throughout a liquid-state hydrogel, but the hydrogel’s solidification transition at body temperature inhibits the drug’s quick release, guaranteeing a longer delivery time. These materials may also produce contractile force and dynamically alter the size in response to temperature variations, accelerating the healing process. As an alternative, scaffolds and crosslinkers that can be broken down by the biochemicals in the wound can be used to create responsive materials that release therapeutic agents [131,132].

Advancements in technology have enabled the development of injectable hydrogel dressings. These dressings are remarkably responsive to temperature fluctuations, ROS, glucose levels, pH balance, and enzymes. Hydrogel dressings can efficiently control their breakdown and drug release by adjusting to different stimuli. This feature makes it possible to treat wounds in a customized way, guaranteeing the most effective achievable healing conditions for all kinds of wounds [133].

Wound healing is affected by temperature-dependent enzymes and their response rate. Temperature is a reliable clinical indication for assessing chronic wounds and identifying classic symptoms. Specialized wound dressings may react or activate differently depending on the temperature. Temperature-responsive hydrogels provide great potential for drug delivery and the healing of damaged tissue. They are often employed to develop responsive systems because of their controllability [134]. Thermoresponsive hydrogels are three-dimensional polymeric networks that change phase in response to temperature. The hydrogel’s dynamic interaction involving water molecules reversibly transforms itself from a liquid to a gel state. Lower critical solution temperature (LCST) is a property of thermoresponsive hydrogels that are liquid at low temperatures and turn to a gel state as the temperature rises. Hydrogels are effective for wound healing because they can gel in vivo at physiologically appropriate temperatures. Polymer chains dissolve in physiological solutions below the LCST, producing a free-flowing liquid. Polymer chains collapse across the LCST by a variety of processes, including micelle packing, coil-to-helix transitions, and hydrophobic interactions. An insoluble gel is produced by the physical bond that forms between the polymer chains. When thermoresponsive polymers reach their upper critical solution temperature (UCST), a single liquid phase remains above it. However, the equilibrium curve indicates that the two phases are separate upon cooling. UCST-polymers exhibit intense supramolecular polymer–polymer interactions, and their behavior is temperature-driven [135,136].

**Figure 6:**
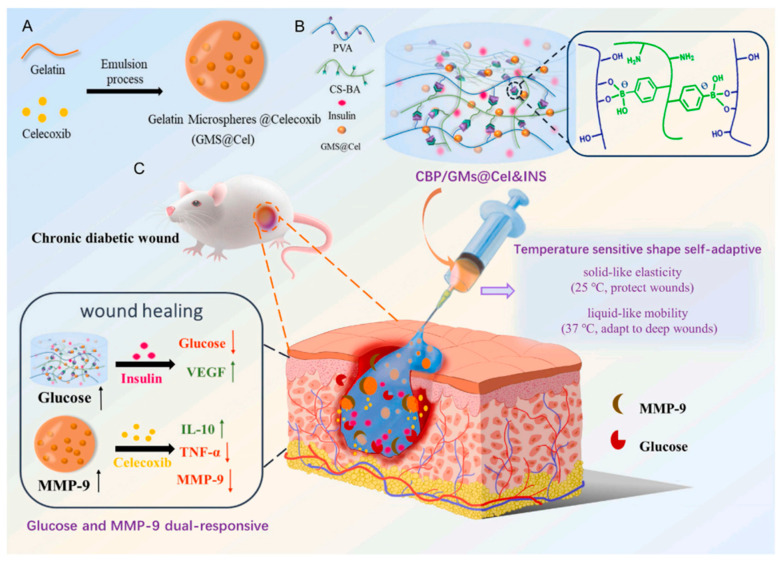
The dual-responsive hydrogel containing glucose and MMP-9 with temperature-sensitive structure and self-adaptive behaviors for the management of chronic diabetic wounds. (**A**) Preparation of gelatin microspheres containing celecoxib (GMs@Cel). (**B**) Preparation of the CBP/GMs@Cel&INS hydrogel and the characteristics of temperature sensitive shape self-adaptive. (**C**) The process of treating chronic diabetic wounds with the CBP/GMs@Cel&INS hydrogel through the glucose and MMP-9 dual-response system. Reprinted from an open-access source [137].

For the treatment of chronic diabetic wounds, a glucose and MMP-9 dual-response temperature-sensitive shape-adaptive hydrogel (CBP@GMs/Cel&INS) was developed with polyvinyl alcohol (PVA) and chitosan grafted with phenylboric acid (CS-BA) by encapsulating insulin (INS) and gelatin microspheres containing celecoxib (GMs@Cel). In the case of chronic diabetic wounds, CBP@GMs/Cel&INS promoted wound healing more effectively through the combined effect of glucose and MMP-9 dual response system and sensitivity to temperature adaptation. On the one hand, the thermosensitive adaptability of the hydrogel ensured that it quickly adjusted to deep wounds and provided mechanical properties to protect wounds. On the other hand, CBP@GMs/Cel&INS on-demand released insulin and the anti-inflammatory drug (celecoxib) under the unique wound environment of high concentrations of glucose and MMP-9. The CBP@GMs/Cel&INS hydrogel has outstanding remodeling, flexibility, and adhesion strength, lowering the danger of infection and boosting drug release efficiency. It also has quick self-healing, extending its duration of use. Wounds treated with CBP@GMs/Cel&INS in a diabetic rat model of a full-thickness skin defect healed more quickly than wounds treated with other groups, exhibiting the most idealized epithelial structure, angiogenesis, and hair follicle regeneration. The synthesis of CBP@GMs/Cel&INS hydrogel and its mechanism are described in Figure 6 [137].

Redox potential is a biological characteristic that can be altered by various circumstances and may change in disease states such as inflammation, cancer, or hypoxia. Therefore, by neutralizing free radicals, preventing free radical chain reactions, and reducing immune system dysfunction, antioxidant hydrogels may be able to lower the excessive ROS in wound sites. Restoring the redox balance and addressing oxidative stress in chronic wounds has been shown to improve appropriate wound healing. There are two primary approaches to synthesizing antioxidant hydrogels: either employing hydrogels as ROS scavenger carriers or utilizing antioxidant molecules as hydrogel precursors to produce hydrogels with intrinsic antioxidant qualities. Research on antioxidant wound dressing materials intended to accelerate the healing process based on available evidence. Hydrogels loaded with natural polyphenols such as gallic acid, tannins, and curcumin can neutralize free radicals. In the case of hydrogels that respond to ROS in the wound environment, the present method involves either incorporating an ROS-responsive block into the central structure of a hydrogel-forming polymer or using polymers with ROS-responsive chain extensions. The hydrolysis or breakage of chemical interactions in the hydrogel with a hydrophobic-to-hydrophilic transition or polymer chain scission is caused by the rise in ROS concentration in the external environment. This eventually results in the regulated release of medications placed into the hydrogel. ROS-responsive hydrogels also generate therapeutic chemicals in reaction to the ROS fluctuations during the healing process, as ROS levels vary according to consecutive phases. Hence, this controlled release of medical substances highlights how responsive they are to the wound’s changing environment [113,138,139].

**Figure 7:**
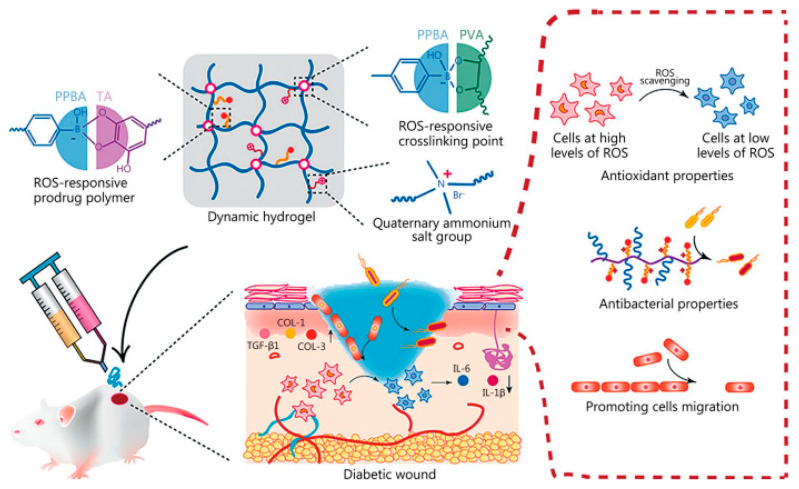
Schematic illustration of the process for reducing inflammation at diabetic wound sites. Reprinted with permission from [140]. Copyright 2022, American Chemical Society.

Ni et al. used the antioxidant characteristics of tannic acid (TA) in conjunction with polyvinyl alcohol (PVA) and phenylboronic acid-modified polyphosphazonitrilene (PPBA) to create a hydrogel (PPBA-TAPVA) that has anti-inflammatory and ROS-responsive capacities. The mechanism of action for hydrogel in diabetic wound management is illustrated in Figure 7. Injectable and self-healing properties that may be customized for irregular deep wounds are provided by the dynamic phenyl borate connection, which enables PPBA-TA-PVA hydrogels to respond effectively to mechanical stresses in continuously moving joint wounds. In vivo tests have shown that PPBA-TA-PVA hydrogel may considerably reduce the inflammatory duration of streptozocin (STZ)-induced rat diabetic wounds and increase wound healing rate when compared to commercially available Tegaderm films. For the management of diabetic wounds that are difficult to heal, wound microenvironment modulation is essential. Excess ROS is thought to be a major contributing factor to the delayed healing of diabetes wounds and can cause infection. In addition to achieving regulated medication release, ROS-responsive hydrogel treatments can lower ROS levels at the wound site, improving the wound microenvironment [140].

**Figure 8:**
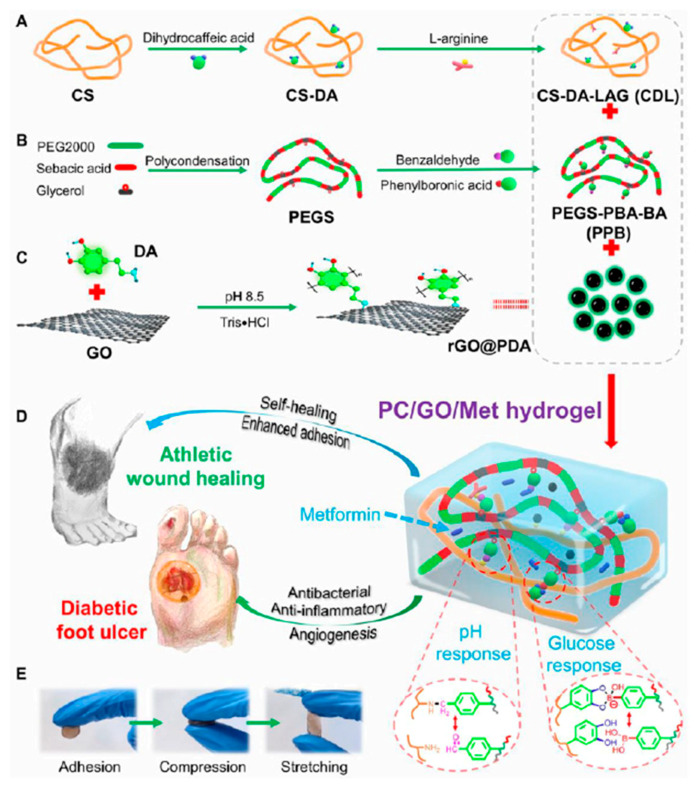
Preparation of (**A**) dihydrocaffeic acid and l-arginine cografting chitosan (CS-DA-LAG), (**B**) phenylboronic acid and benzaldehyde difunctionalized polyethylene glycol-co-poly(glycerol sebacic acid) (PEGS-PBA-BA) and (**C**) polydopamine coated rGO (rGO@PDA). (**D**) The schematic diagram of structure, pH and glucose responsive mechanism of PC hydrogel and its application in diabetic foot ulcers and athletic wound healing. (**E**) Representative pictures of PC hydrogel for adhesion, compression, and stretching. Reprinted with permission from [144]. Copyright 2022, American Chemical Society.

pH serves as an indicator of the wound’s state, and pH variations can be utilized to forecast whether a wound will heal or deteriorate. The pH levels vary according to the skin’s condition: healthy skin usually has a slightly acidic pH (5–6), acute wounds have a pH of about 7.4, and chronic wounds have a more alkaline pH of 7.3–10, in part because of the presence of bacterial colonies that are growing. Both bacterial infection and colonization, which are frequent features of chronic wounds, may be impacted by these pH variations. Wound healing can be accelerated by properly controlling the pH of the wound during the various healing stages. Restoring the acidic conditions in chronic wounds can improve adipose tissue metabolism and lessen microbial colonization on the skin’s surface. However, keratinocyte and fibroblast migration and proliferation favor a slightly more alkaline environment with a pH of about 8.3. An alkaline environment, which is seen during wound hyperplasia and remodeling, can promote cell proliferation and skin remodeling, whereas an acidic environment, which occurs during the early stages (hemostasis and inflammation), can prevent bacterial infection and encourage vascular regeneration [141,142,143]. Liang et al. reported a multifunctional adhesive, antibacterial, antioxidant, and conductive phenylboronic acid and benzaldehyde bifunctional polyethylene glycol-co-poly(glycerol sebacic acid)/dihydrocaffeic acid and Larginine co-grafted chitosan (PEGS-PBA-BA/CS-DA-LAG, denoted as PC hydrogel dressing that has strong self-healing capacity and pH/glucose dual-responsive drug-release characteristics) was developed for the reconstruction of athletic diabetic foot wounds. The rheological and mechanical properties, self-healing ability, and pH/glucose dual-responsive metformin release ability provided by double dynamic bonds were all confirmed through the results obtained. Cationic antibacterial properties provided by L-arginine, tissue adhesion, blood coagulation, in vivo hemostasis and antioxidant properties provided by catechol structure, and biocompatibility of PC hydrogels were other characteristics provided by the PC hydrogel. The wound closure ratio, re-epithelialization ratio, and regeneration of blood vessels and follicles were all evaluated to show that PC hydrogel enhanced healing of the type II diabetic foot by lowering inflammation and enhancing angiogenesis. Figure 8 depicts the whole approach for developing a PC hydrogel for diabetic foot wound healing [144].

Near-infrared (NIR) radiation (700–1000 nm) has more tissue transparency than other wavelengths, making it a suitable candidate for designing light-responsive hydrogels with precise control. Photothermal and upconversion effects are the two main mechanisms that have been studied in NIR-responsive hydrogels employing NPs as transducers. The upconversion effect describes a process where the successive absorption of multiple photons in NPs results in light emission at a shorter wavelength compared to the excitation wavelength, including the conversion of NIR light to UV light. The photothermal effect is the discharge of vibrational energy (heat) when an NP photosensitizer is excited with specific band light, demonstrating the conversion of light to heat [145]. Materials containing NIR-responsive additions may cause the hydrogels to decompose thermally, releasing the active ingredients inside their structure. Heating the structures may result in a more rapid and controlled drug release when it is enclosed in a hydrogel structure and takes the form of NIR-sensitive nanoparticles. Increased drug permeability can be attributed to modifying the hydrogel’s structure. When subjected to near-infrared light, these photothermal agents—such as carbon nanomaterials, silver nanoparticles, or gold nanoparticles—can generate heat, facilitating medication release [146].

Stimuli-responsive hydrogels can be characterized by the stimulus they respond to and the active component they incorporate. The resultant biomaterial’s final physiochemical and therapeutic qualities are determined by the hydrogel matrix and the loading agent; these features are succinctly outlined in Table 3. Most active components in this type of hydrogel are antibacterial compounds, with a focus on non-antibiotic molecules. The basis for this development is the search for replacement medications that many bacterial strains have become resistant to. Polyphenols, antibacterial polypeptides, and silver nanoparticles are now the most employed materials to produce stimuli-responsive hydrogels containing non-antibiotic compounds [91,111,147].

Hydrogels containing phages have been used to treat bacterial wound infections. A distinct zone of suppression on the *S. aureus* bacterial lawn was demonstrated by Phage K, which was prepared in a hyaluronic acid methacrylate (HAMA)/agarose hydrogel system [165]. Additionally, the formulation’s in vitro effectiveness has been shown by the ability of temperature-sensitive Poly(N-isopropylacrylamide-co-allylamine) (PNIPAMco-ALA) hydrogel bonded to non-woven polypropylene to distribute infectious phage K and create a bacterial zone of clearance (Table 4) [166]. Another study found that after 6 h in vitro, treatment with a PVA-SA hydrogel-based membrane containing the phages MR10, Kpn5, and PA5 (all at a reported Multiplicity of Infection—MOI of 10) led to a 6 log reduction in *S. aureus* biomass, a 6.37 log decrease in Klebsiella pneumoniae, and a 4.6 log diminish in *P. aeruginosa* biomass (Table 4) [167]. In mice with an *S. aureus* burn wound infection model, treatment with MR10 hydrogel led to faster wound healing than the untreated control, especially when minocycline was added. The skin layers, sweat glands, and hair follicles of mice undergoing dual-agent treatment completely regrew on day 14, resembling that of normal mouse skin. Phage hydrogels accelerated wound healing and markedly decreased mortality in a mouse burn wound infection model. In contrast to other antimicrobials like silver nitrate and gentamicin, a study by Kumari et al. [168] found that therapy with phage Kpn5 in HPMC hydrogel (MOI = 200) increased the rate of survival of Klebisella-infected animals (Table 4). On day 1, 87% of the mice in the non-treated group survived compared to all the mice in the phage hydrogel-treated group. On day 7, the phage-treated group exhibited the highest level of protection (63%) compared to the untreated group (0%). When phages and antibiotics are used together, the survival rate may be significantly raised.

Antibacterial peptides (AMPs) are naturally occurring short polypeptides (~12–50 amino acid residues) with wide antibacterial activity against bacteria, viruses, and fungi. AMPs have become a popular alternative to traditional antibiotics due to their superior antibacterial capabilities and low risk of resistance development [173]. Nanoparticle-based AMP delivery is a promising treatment for bacterial infections due to its capacity to protect, stabilize, and deliver AMPs to specific areas.

Primo et al. [174] recently created AMP Ctx(Ile21)-Ha-conjugates with rifampicin (RIF)-loaded NPs to enhance resistance, physiochemical stability, and antibacterial activity. According to the study’s findings, AMP-conjugated RIF-loaded chitosan-based NPs effectively delivered the anti-tuberculosis medication RIF (with 100% bioavailability) into macrophages. Additionally, these NPs demonstrated outstanding antibacterial activity against clinically obtained multi-resistant strains of the bacillus *Mycobacterium tuberculosis* (MTB), indicating that AMP-nanocarrier systems may be able to overcome multi-drug resistance in bacteria. Li et al. [175] investigated the potential use of antimicrobial peptides in cutaneous wound healing by developing an injectable in situ gel-forming device made of thermosensitive hydrogel and nanoparticles loaded with human antimicrobial peptides 57 (AP-57). The thermosensitive hydrogel with nanoparticle encapsulation was made from biodegradable poly (L-lactic acid)-poly (L-lactic acid)-PLLA-L35-PLLA. This study’s hybrid hydrogel-based delivery method, which has minimal toxicity and high antioxidant properties, showed prolonged in vitro release of AP-57. Additionally, utilizing a full-thickness dermal defect model, the in vivo investigations employing the AP-57 nanoparticles–hydrogel system demonstrated improved granulation tissue development, collagen deposition, and angiogenesis in addition to considerable wound healing with a wound closure of around 96.78 ± 3.12%. Gram-positive *S. aureus* infections and Gram-negative *P. aeruginosa* infections were successfully treated in a different investigation using a hydrogel/liposome system for the photothermally driven release of AMPs. Here, AMP, IRIKIRIKCONH2 (IK8), and liposomes and gold nanorods (AuNRs) were co-loaded into a poly (ethylene glycol) (PEG) hydrogel to limit infection. Photothermal triggering of IK8 with laser irradiation at 2.1 W cm^−2^ for 10 min resulted in heating to 55 °C and improved antibacterial activity. *P. aeruginosa* and *S. aureus* populations were reduced by approximately 6- and 7-log because of the induced release of IK8 at this laser intensity. Furthermore, increasing the laser intensity to 2.4 W cm^−2^ and heating to 60 °C resulted in a 5- and 2-log reduction in viable *P. aeruginosa* and *S. aureus* populations [176]. Another study examined the synthesis of peptide-based hydrogel composites with photo-generated silver nanoparticles (AgNPs) using a one-pot green synthetic method. The composites were made with hydrogel stabilizer and highly aromatic tripeptide hydrogels (hgel) containing AgNPs, both with and without honey as a tensile strength enhancer. AgNPs were photosynthesized inside peptide hydrogels in either the presence or absence of honey, and a green material was employed to cap the AgNPs, increasing the hydrogel’s mechanical characteristics. Due to their ability to absorb sunlight and create electron radicals, the gel’s peptide building blocks’ aromatic moieties were essential to the reduction in silver ions. It was found that adding honey to the composites reduced the size of the AgNP particles and created uniform NPs. It also enhanced the gel’s mechanical qualities and swelling capacity, both of which are critical characteristics for upcoming uses, including wound healing. According to the findings of the antibacterial investigations, the minimum inhibitory concentration for *S. aureus* ATCC 25923 and the methicillin-resistant *S. aureus* isolation was considerably reduced by AgNPs/hgel and AgNPs/hgel-honey when compared to hgel, Ag, and Ag/honey [177].

## 8. Applications of Smart Hydrogels in Real-Time Monitoring

Traditional wound management often divides wound healing and monitoring. The rate of wound healing is evaluated during routine dressing changes after the wound has been cleaned and medicated. In addition to causing secondary harm to the patient’s wound, this treatment mode may result in postponed dressing changes because it does not track wound healing in real-time. Thus, one of the biggest challenges for therapeutic tissue regeneration is how to monitor wounds in real-time while they heal. An emerging study area is smart composite hydrogel-based sensors tracking wound healing [178,179,180]. Hydrogel sensors are highly biocompatible, making strong connections with biological tissues and minimizing the possibility of immune responses. As a result, hydrogel sensors present opportunities for individualized healthcare and are appropriate for long-term in vivo monitoring. Because hydrogel sensors are highly sensitive to even the smallest physiological changes, they can monitor various physiological indicators in real-time, including heart rate and blood sugar, and provide accurate data for prompt responses. Hydrogels’ durability allows for dependable functioning inside the biological environment for lengthy periods of monitoring chronic illnesses or situations needing continuous surveillance. With the ability to detect numerous physiological factors or biomolecules at once, hydrogel sensors can be created as multipurpose monitoring tools that exhibit a wide range of diagnostic and monitoring capabilities in a variety of disease domains, including wound healing [181,182,183,184].

**Figure 9:**
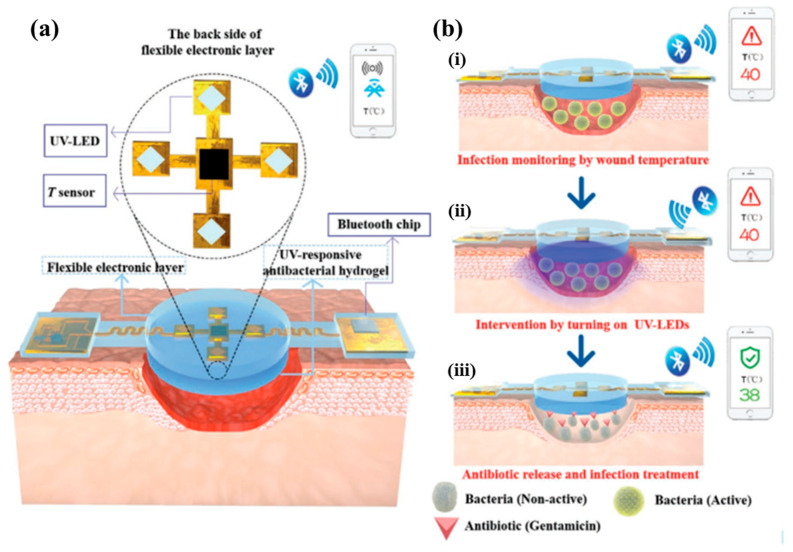
Schematics of the architecture and operating principles of the smart flexible electronics-integrated wound dressing. (**a**) System integrating a polydimethylsiloxane-encapsulated flexible electronic layer and an UV-responsive antibacterial hydrogel. (**b**) Conceptual view of the integrated system for infected-wound monitoring and on-demand treatment: (**i**) real-time monitoring of wound temperature and providing an alert of hyperthermia caused by infection; (**ii**) turning on UV-LEDs to trigger the release of antibiotics; (**iii**) infection inhibition by the released antibiotics, resulting in decreased wound temperature. Reprinted from an open-access source [185].

An essential factor in wound detection is wound temperature. A high temperature indicates the possibility of infection, inflammation, or engorgement of the incision. A reduction in wound temperature may indicate lower collagen deposition and fewer late-phase regenerative inflammatory cells and fibroblasts. Lin et al. [131] created a smart wound dressing that includes drug delivery channels, temperature sensors, and reservoirs to monitor wound healing and administer medication. Temperature sensors, diffusive drug reservoirs, and nondiscursive drug-delivery routes (made of plastic tubes) were patterned into the hydrogel matrix to produce the smart dressing. The as-prepared intelligent dressing can control drug release and identify wounds. The drug solution can be manually transferred to the appropriate drug reservoir via the non-diffusion drug delivery channel when the sensor indicates that the temperature at a specific location rises above a threshold (for example, 35 °C), after which it can gradually and carefully diffuse out of the hydrogel matrix. Pang et al. developed an intelligent, flexible, electronics-integrated dressing that can monitor the temperature of the wound in real time to detect infections early in chronic wounds and treat them as required by releasing antibiotics when exposed to UV light. Flexible polydimethylsiloxane (PDMS) was used to encapsulate the entire electronic system to guarantee its high biocompatibility, permeability, and transparency levels. The lower layer was a 3 mm-thick UV-responsive antibacterial hydrogel in which gentamicin (GS) was covalently grafted into a polyethylene glycol (PEG)-based hydrogel via a UV-cleavable linker and released upon irradiation at 365 nm; an integrated temperature sensor continuously recorded wound temperatures, which were then sent in real-time via Bluetooth to a smartphone, and UV-LEDs were utilized to regulate the in situ release of antibiotics remotely (Figure 9). In vitro tests revealed that the integrated system has outstanding flexibility, excellent compatibility, high monitoring sensitivity, and long-term durability in humid conditions. By developing an infection model in a Bama mini pig, it was confirmed that the integrated system could track the condition of the wound in real time, anticipate infection early, and treat them as needed [185].

**Figure 10:**
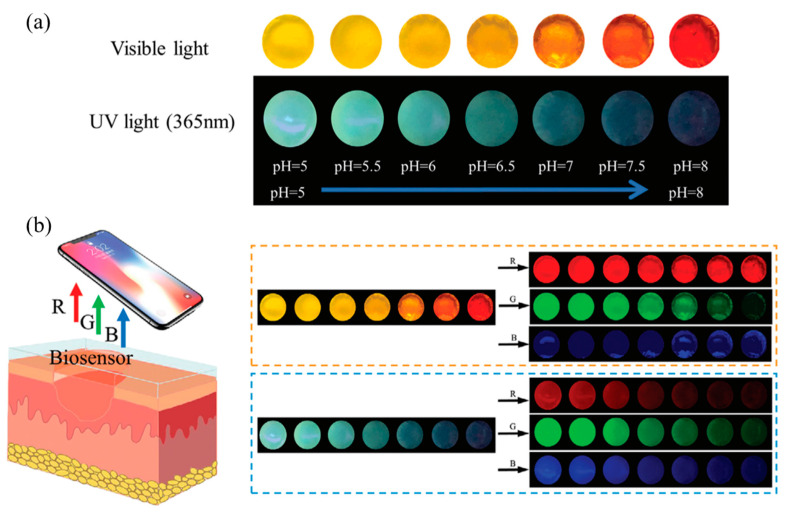
(**a**) The hydrogels’ pH sensitivity operates in the presence of UV light and sunshine. (**b**) Utilizing smartphones to read and track the pH of hydrogels. RGB pictures of PAM-QCS-C-P hydrogels exposed to UV and visible light at various pH levels. Reprinted with permission from [186]. Copyright 2021, Wiley Materials.

The pH value, which indicates the level of tissue regeneration and wound infection, is a crucial monitoring signal during the wound healing process. A natural defense against microbial illness is provided by normal skin with a pH of 4–6. In contrast, the action of bacteria and their enzymes causes the pH of the wound to rise to 7–8 after infection. Quaternary ammonium chitosan (QCS) and polyacrylamide (PAM) were initially physically crosslinked by Zheng et al. [186] to create PAM-QCS hydrogels. Diffusion hybridization was then used to load the CQDs and pH indicator (phenol red) into the PAM-QCS gel. Excellent biocompatibility and antibacterial capabilities are conferred by PAM and QCS, respectively, within the produced PAM–quaternary ammonium salt chitosan–CQD–phenol red hydrogels (PAM-QCS-C-P hydrogels). Through physical entanglement, the two create a network that is cross-linked. A two-color monitoring system based on visible color and fluorescent signals was built using pH-sensitive CQDs and phenol red in response to the wound condition’s pH change (5–8) (Figure 10a). Zheng and colleagues greatly increased the accuracy of pH monitoring by using a smartphone to take images of PAM-QCS-C-P hydrogels and convert them into RGB signals for remote processing. Under visible light, increasing the pH value improves the R-value of the PAM-QCS-C-P hydrogel while decreasing the G value and maintaining the B value. The signal that corresponds to the pH value is G/B. Under UV light, the R, G, and B values of PAM-QCS-C-P hydrogels diminished with higher pH levels. The hydrogels had outstanding hemostatic, skin-adhesive, moisture-retaining, and antibacterial properties, which worked together to promote wound healing effectiveness (Figure 10b).

**Figure 11:**
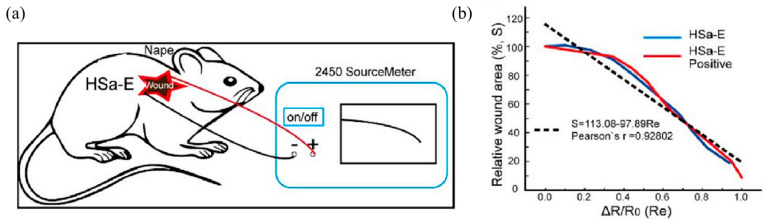
(**a**) The wound healing and monitoring experiment’s diagram. (**b**) Resistance and relative wound area correlation curves in the HSa-E and HSa-E/Positive groups. Reprinted with permission from [187]. Copyright 2022, Elsevier.

The degree of healing is typically indicated by the wound area. By identifying the wound region, some researchers attempt to track the healing process. For instance, Chen et al. [187] used sodium alginate (SA) and chitosan quaternary ammonium salt (HACC) as raw ingredients to create HSa hydrogels using straightforward processes. The electrostatic connection between the positive charge of the amino group in HACC and the negative charge of the carboxylic acid group in SA generates a “Magic Cube”-like structure that may be transformed arbitrarily, like a Rubik’s cube. Free Cl^−^ gives the HSa hydrogel its high electrical conductivity (1.14 × 10^−3^ S cm^−1^) and a positive linear relationship between the resistance change and the area change. The HACC-SA (HSa) hydrogel’s characteristics enable it to be used to measure the area of the wound to evaluate wound healing. After applying the hydrogel to the mouse’s wound, the researchers measured the hydrogel’s resistance change using a digital meter (Figure 11a). The viability of using HSa hydrogel for wound detection is demonstrated by the positive linear association between the relative wound area change and the measured relative resistance change, ΔR/R0 (Figure 11b). Furthermore, Chen and coworkers looked at HSa hydrogels’ capacity to accelerate wound healing. On days 6, 9, and 12, the HSa group’s wound healing performance outperformed the control groups, indicating that HSa hydrogel can effectively stimulate wound healing in vivo.

Impedance detection is a method for assessing the skin’s physiological state, especially in closed wounds or subcutaneous tissue injury cases. Ischemia and tissue damage are linked to pressure ulcers, which raise conductivity because dead cells discharge ion-rich cytoplasm. As a result, skin impedance measurement enables early ulcer diagnosis. A flexible gold electrode array for impedance mapping was created by Swisher et al. [188]. The magnitude and phase angle spectrum were recorded when the sensor was applied to a rat model of tissue injury caused by pressure. This indicates a strong correlation between the frequency spectra of impedance recordings and the condition of the underlying tissue in various animals and wound types. Histological cross-sections taken at different intervals during the investigation support the theory that modifications to tissue structure and cell membranes cause the observed variations in impedance. All skin samples collected following the 3 h magnet exposure period exhibited signs of ulceration, whereas none from the 1 h application group did. These histology data were in line with the anticipated changes in impedance brought on by modifications in tissue structure. The results confirmed that an automated, non-invasive “smart bandage” for pressure ulcer early detection is feasible, enhancing patient care and results. A promising method for tracking wound conditions and accelerating healing at the same time is the combination of surgical sutures and biosensors [188,189].

**Figure 12:**
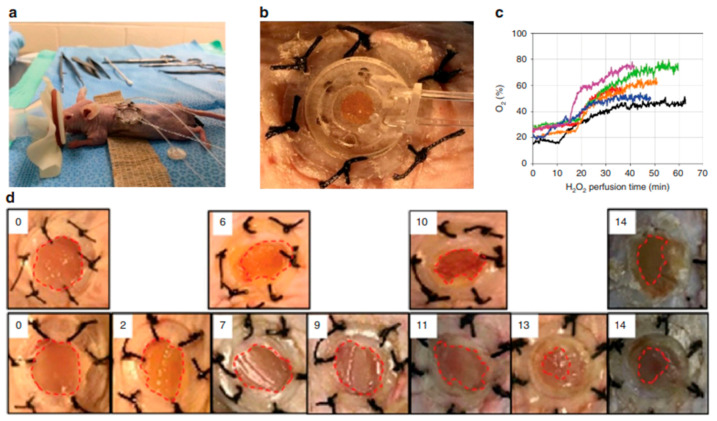
In vivo testing of smart dressing devices. (**a**) Surgical equipment. (**b**) A close-up of the equipment demonstrating the formation of oxygen bubbles during H_2_O_2_ perfusion. (**c**) Measurements of wound oxygen taken during in vivo H_2_O_2_ perfusion of devices. (**d**) Evolution of wound healing in SKH1 mice: days 0, 6, 10, and 14 in the Integra control and days 0, 2, 7, 9, 11, 13, and 14 in the oxygenated wounds. Reprinted from an open-access source [190].

Suboptimal wound bed oxygenation is one of the most important and curable wound treatment aspects; yet, current oxygenation technologies lack the capability for simultaneous oxygen assessment and administration in a wearable platform. Ochoa et al. proposed a low-cost, paper-based, biocompatible, and flexible platform for continuously delivering and monitoring oxygen in wounds. The platform uses recent advances in the manufacture of flexible microsystems, such as using paper as a substrate and inkjet printing, a scalable manufacturing process. It has been shown how the oxygenation patch works, raising the oxygen content in a gel substrate by 13% (5 ppm) in only one hour. The platform can detect oxygen levels between 5 and 26 parts per million. The patch’s biocompatibility and capacity to double or triple the oxygen content in the wound bed to clinically meaningful levels are demonstrated by in vivo tests. Mice were used to assess the devices’ functionality, biocompatibility, and wound-healing effectiveness. According to wound measurements, the first 10–20 min of the first equilibration phase saw a 0–5% rise in wound oxygen above ambient (Figure 12c). Oxygen concentrations increased more dramatically by an additional 25–45% during the next 40–50 min. Digital pictures of the wounds were obtained to calculate the wound area, and oxygen treatments were repeated every day for up to 60 min using modern equipment. Because H_2_O_2_ reacts with the wound bed during perfusion and penetrates the paper barrier, the wound healing rate of oxygenated wounds was somewhat slower than that of non-oxygenated wounds (Figure 12d) [190].

Pressure sensors can promptly warn of excessive mechanical stress on the tissue to prevent ulcers. Diabetic foot ulcers (DFUs) are thought to occur in 19% to 34% of diabetic people. Shoes with pressure sensors are recommended by worldwide recommendations to stop DFUs from happening again. Pressure monitoring can alert patients to release elevated pressures and stop ulcer formation, whereas impedance sensing offers early warning as soon as tissue damage occurs. Piezoresistive pressure sensors have also gained popularity due to their ease of manufacture and operation. However, the operational range of piezoresistive materials is limited since they often show a negative resistance fluctuation against external forces. Wu et al. [191] used laser-scribed graphene to develop a positive piezoresistive plantar pressure sensor to overcome this restriction. Random fractures between graphene layers developed under the given pressure, leading to a dramatic increase in resistance. The sensor’s sensitivity of 12.3 kPa^−1^ at 200 kPa and resistance change rate of >360,000% were made possible by the positive resistance pressure correlation feature [101]. Ulcers are not produced by sudden pressure but, rather, grow gradually. Abbott et al. [192] investigated the effectiveness of a foot pressure sensor with continuous feedback and self-direct adjustment modules (SurroSense Rx, Orpyx Medical Technologies, Canada) in diabetic patients through randomized proof-of-concept clinical research. Instead of concentrating on the maximum peak plantar pressure, the sensor captured the static high pressure and alerted the user. In contrast to the control group, they observed a lower ulceration rate in the intervention group, indicating that static pressure has a direct role in developing planar ulcers. For more precise and effective ulcer therapy, several types of pressure signals with additional biomarkers must be integrated.

## 9. Limitations and Challenges of Hydrogels

Exploring possibilities for ideal bioinks is an important field of study in wound dressing and tissue engineering, focusing on developing materials that meet a wide range of parameters. Enhancing cell adhesion, assuring efficient printability, and promoting cell migration are some of the requirements. This effort highlights the current reliance on certain materials, exposing a significant deficiency in the variety of hydrogels designed for particular uses, as evidenced by several research studies [193,194,195].

Although progress has been made, developing hydrogels to mirror the mechanical characteristics of actual tissues remains an important task. These characteristics include durability, electrical conductivity, and strength. In addition, because hydrogels are inherently viscoelastic, they provide challenges concerning aging and creep, where ongoing tension or strain may result in time-dependent deformation. The hydrogel’s capacity to maintain cell attachment, migration, and proliferation—all essential for efficient tissue repair and regeneration—may be compromised by this behavior. To overcome these obstacles, a well-thought-out strategy is needed to balance biological and mechanical properties to accommodate certain cell types and their roles while guaranteeing the materials’ durability in clinical settings [196,197,198,199].

Another significant challenge is accurately regulating the behavior of stem cells, including their differentiation, proliferation, and integration with bodily tissues—all of which are essential for the processes of regeneration and healing. Promoting cell migration and proliferation, mimicking the signals of the natural extracellular matrix, and blending in with the body’s tissues constitute each part of this issue. The effectiveness of hydrogel-based dressings partly depends on controlling immune reactions to them and improving their bioactivity [194,200,201]. Hydrogels are constantly being improved to achieve superior properties in regulated and targeted drug delivery. The goal is to create hydrogel dressings that provide a moist environment and precisely dispense growth factors and other therapeutic compounds or release drugs to promote tissue regeneration and re-epithelization. In regenerative medicine, the objective is to improve patient outcomes by increasing therapy efficacy while reducing side effects [202,203].

## 10. Future Perspectives in Hydrogel Research

Standard wound care procedures involving dressings, topical therapies, and skin grafts can fail to address the complex characteristics of chronic wounds. Traditional dressings are unable to promote tissue regeneration, provide an optimal healing environment, or effectively control exudate. Skin grafts and advanced wound care products may not be appropriate for all patients since they involve graft rejection, limited availability, and high pricing. Moreover, these approaches generally have little impact on the root causes of chronic wounds, which might include insufficient circulation or chronic inflammation [204,205,206].

The concept of personalized medicine is acquiring interest in wound care, with 3D printing enabling the development of tailored and on-demand wound healing treatments. Hydrogels, biodegradable polymers, and bioinks represent some of the materials used in biomedical 3D printing. Personalized techniques entail adjusting treatment plans to each patient’s particular needs, including their tissue type, wound form, and healing demands. 3D printing makes it possible to produce unique wound care items for each patient, such as scaffolds and dressings that are precisely the proper size to match the wound’s characteristics. Additionally, on-demand treatments allow for fast reaction and flexibility since wound care solutions are produced in real-time in response to urgent patient needs [207,208]. Three-dimensional bioprinting facilitates the formation of vascular networks within the printed tissue. Eventually, this vascularization helps the healing procedure for venous ulcers by guaranteeing that the regeneration cells receive an appropriate blood supply. The methodical creation of complex vascular networks within the bioprinted tissue is made possible by 3D bioprinting, which presents a novel strategy. The vascular architecture necessary to maintain cellular regeneration is recreated by the meticulous stacking of bioinks, which include growth hormones, biomaterials, and living cells [209].

A study by Mohd Yazid Bajuri and associates [210] evaluated the impact of fibrin gel-stabilized 3D-bioprinted autologous adipose tissue grafts on DFUs. A tertiary institution specializing in diabetic wound care hosted this single-arm pilot research. The main objective was to achieve complete healing in 12 weeks for ten individuals with DFUs. Notably, during the designated 12-week interval (at 2, 4, 5, 10, and 12 weeks), seven of ten patients experienced complete DFU healing. Over time, the reduction in wound size showed a notable and ongoing decrease. According to the study’s findings, using fibrin gel as a supporting framework in conjunction with autologous adipose tissue grafts made by 3D bioprinting enables superior skin restoration and encourages efficient wound healing. Additionally, no adverse effects were noted during the trial.

The use of computational simulations and modeling approaches marked the beginning of the integration of AI in hydrogel research in the 1990s and early 2000s. Researchers began simulating hydrogel behavior under various situations using computer techniques to predict swelling characteristics, mechanical reactions, and drug release kinetics. Concepts from continuum mechanics, including constitutive equations, kinematics, and balance laws, form the foundation of hydrogel theoretical modeling. An appropriate example is the Flory–Rehner theory, which describes the swelling equilibrium of gels. As computing capabilities improved, the computational science paradigm became increasingly popular. Both macro- and micro-scale simulations, including those based on the volume and finite element approaches, are now feasible. AI provides an innovative approach to address the difficulties with hydrogel performance and properties in hydrogel development by utilizing optimization methods, predictive modeling, and data-driven insights. These difficulties have many aspects and involve modifying hydrogels for specific uses, enhancing their mechanical and chemical properties, and managing the intricate interactions between various material properties. Above all, AI accelerates the process of developing novel hydrogel compositions. Conventional approaches frequently require much trial-and-error testing, which takes much time and money. On the other hand, AI-powered algorithms can anticipate material qualities, assess large datasets quickly, and suggest the best compositions. In the field of hydrogels, where materials must satisfy exacting requirements for applications in biology and medicine, expediting the research process is very important [211,212].

The hydrogel research community acknowledged that artificial intelligence (AI) could completely transform material discovery as machine learning (ML) techniques improve. The application of machine learning techniques has become popular. These methods allowed researchers to precisely predict the behavior of hydrogels, produce structure–property correlations, and examine enormous datasets. Producing hydrogels that precisely transport medications, AI algorithms can evaluate patient-specific data, including metabolism rates, genetic information, and medical histories. AI-driven hydrogel formulations, for example, can modify medication release rates in cancer treatment according to the tumor’s reaction to therapy, limiting side effects and increasing efficacy. For instance, they can forecast how a hydrogel will react to variations in pH, temperature, or biological variables by swelling, breaking down, or releasing drugs. The best mix of crosslinkers, polymers, and additives can be found using AI-driven material design to produce hydrogels with mechanical, thermal, and chemical characteristics. This accelerates the production of hydrogels suited for uses like tissue scaffolds, contact lenses, and wound healing. AI-guided high-throughput screening makes it possible to assess enormous libraries of hydrogel formulations. This shortens the time required to introduce novel materials to the market by speeding up the discovery of hydrogels with desired qualities. As a result, this period witnessed a dramatic change from conventional empirical methodologies to data-driven approaches, which allowed for quicker and better-informed hydrogel design decision-making [211,213,214].

## 11. Conclusions

Chronic wounds are a major concern in healthcare, affecting millions of people globally, particularly those with underlying diseases, including diabetes and obesity. By describing the prevalence of chronic wounds and their burden on healthcare systems, this review has brought attention to the complex nature of managing these wounds. Advanced wound treatment products, particularly hydrogels, are becoming well-known as cutting-edge treatments made to handle these challenging issues successfully. Examining several kinds of chronic wounds, including pressure ulcers, diabetic foot necrosis, and venous leg ulcers, reveals both shared underlying causes and particular problems. Each variety provides unique clinical challenges exacerbated by infection, biofilm formation, and patient-specific considerations such as comorbidities.

Hydrogels have been recognized as a promising alternative for chronic wound care due to their capacity to retain moisture, induce cellular migration, and provide antibacterial properties. The benefit of hydrogels over conventional wound dressings is further supported by clinical applications that show notable improvements in patient outcomes. While research is moving towards integrating digital health solutions for comprehensive wound management, hydrogel technology innovations, including stimuli-responsive and drug-loaded varieties, continue improving their functionality. Stimuli-responsive hydrogels are adaptable in the wound environment, releasing microbial compounds or introducing bioactive substances such as growth hormones, antibiotics, or peptides to promote healing in real time. Controlled delivery of these substances allows for maintaining ideal concentrations at the wound site for prolonged periods, which minimizes patient discomfort and the need for frequent dressing changes. These formulations have dramatically increased healing rates, especially in resistant wounds. Attention to personal customization based on wound characteristics and patient-specific parameters is essential to guarantee that hydrogel-based therapies are as effective as possible. One concept is to develop hydrogels that respond to specific wound indicators (such as pH, inflammatory cytokines, or glucose levels) observed in individual patients or to use patient wound exudate to evaluate the molecular environment and choose appropriate hydrogel components. A viable strategy for this direction may involve integrating particular medications, such as growth factors, antibiotics, or anti-inflammatory treatments, according to the patient’s wound type and stage (e.g., VEGF for angiogenesis or TGF-β for tissue regeneration). Personalized drug-release kinetics in engineered hydrogels will provide consistent therapeutic levels throughout time, reducing the necessity for frequent dosing. Moreover, adding patient-derived cells to hydrogel formulations—such as adipose-derived stem cells (ADSCs) or mesenchymal stem cells (MSCs)—will support immunomodulation and tissue regeneration. A new path in the hydrogel area is represented by integrating several hydrogel types inside a single structure for targeted therapy and using 3D bioprinting to produce patient-specific hydrogel patches that fit the depth and shape of the wound. These methods improve healing results, lower complications, and increase patient satisfaction.

According to the current state of the art, further studies in the field are expected to deepen the current research and advance the proposed emerging solutions from in vitro/in vivo testing to more detailed testing stages. Conducting robust clinical trials will demonstrate the efficacy and safety of the hydrogels, building confidence among healthcare regulators. The transition from the laboratory to manufacturing at the industrial level addresses key challenges such as hydrogel scalability, functionality, and optimization of manufacturing costs. Utilizing source polymers and bioactive agents that are widely available and sustainable, and improving the hydrogel synthesis, will lower production costs without compromising their functionality. Manufacturing methods such as 3D printing or bulk mixing will allow large-scale production while maintaining quality. Partnerships with pharmaceutical companies will help bring products to the market.

Although hydrogels promise to revolutionize soft and hard tissue treatments, their successful application necessitates overcoming several challenges through ongoing research, teamwork, and regulatory compliance. Numerical simulations foster collaboration between computational and experimental approaches, especially for chronic wound treatment, by providing a virtual playground for customizing, improving, and fine-tuning hydrogel properties. New hydrogel formulations with specialized functions will be discovered because of multidisciplinary innovation fostered by partnerships between chemists, materials scientists, and AI specialists. Some feasible solutions could be represented by integrating digital health technologies that will overcome the current limitations of hydrogels. A suggestive treatment stands for introducing sensors into hydrogels to monitor wound conditions in real time and provide feedback to patients or clinicians. Mobile application integration, which implies pairing hydrogel treatment with applications for wound tracking, telemedicine consultations, and combining hydrogels with wearable technologies, is projected to improve patient outcomes and provide continuous care and monitoring.

To conclude, hydrogels hold tremendous potential for improving management strategies for chronic wounds ranging from diabetic foot necrosis to decubitus ulcers, enabling the development of performant multifunctional dressings. Hydrogel-based therapeutic approaches can be advanced to more elevated testing stages through further interdisciplinary studies and eventually reach clinical settings as personalized wound care solutions.

## Figures and Tables

**Table 1:** Comparison between hydrogel types in terms of biocompatibility and effectiveness.

Type of Hydrogel	Biocompatibility	Cost	Effectiveness	Advantages	Disadvantages/Limitations	Refs.
Natural hydrogels	High	Low	Excellent for wound healing and tissue engineering.	High biocompatibility and biodegradability. Mimics natural extracellular matrix. Support cell adhesion and growth. Renewable and environmentally friendly. Intrinsic bioactivity (cell signaling, antimicrobial properties in some cases). Easy to crosslink with mild conditions (ionic or thermal processes)	Low mechanical strength and elasticity. Limited control over degradation rate. Batch-to-batch fluctuations brought on by natural sources. Susceptible to microbial contamination or degradation without proper treatment. Short shelf-life. Could require chemical modifications to enhance stability and performance.	[58,59,60,61,62,63,64]
Synthetic hydrogels	Moderate	Moderate	High mechanical stability and tunable properties. Less bioactive without modification.	Stable and reproducible. Versatile in drug delivery and tissue regeneration. Easy to tailor physical and chemical properties. Minimal degradation. Can be modified for controlled release or specific bioactivity.	Limited bioactivity without modification. Potential cytotoxicity or inflammatory response depending on the polymer type. Some polymers are non-biodegradable (PHEMA). Can require chemical crosslinking, which can be toxic. Hydrophobicity involves surface modification to improve interactions with biological systems.	[56,65,66,67,68,69]
Hybrid hydrogels	High	High	Combines biocompatibility, bioactivity, and mechanical strength from natural and synthetic hydrogels. Suitable for advanced biomedical applications (drug delivery, regenerative medicine, and tissue scaffolds).	Customizable properties to suit specific applications. Can achieve controlled degradation and release rates. Suitable for multifunctional uses, including 3D bioprinting and personalized medicine.	High manufacturing complexity and cost. Requires advanced synthesis technologies. Potential compatibility issues between natural and synthetic components. Limited scalability due to sophisticated production methods. Can require modification strategies in order to enhance biocompatibility.	[70,71,72,73,74,75]
Smart hydrogels	High	High	Highly effective in controlled drug delivery and tissue engineering due to their responsiveness to stimuli. Effectiveness varies based on the type of stimuli (pH, temperature, light, ROS, etc.)	Responds to specific stimuli, enabling precise control over drug release, swelling, or structural changes. Can adapt dynamically to changing environment (i.e., temperature-sensitive for wound healing). Enable innovation in targeted therapies and personalized medicine.	Limited scalability for industrial or large-scale production. Requires specialized materials and techniques. Stability and performance can degrade over repeated stimuli cycles. Modeling in vivo release profiles is necessary before commercialization.	[76,77,78,79,80,81]
Ionic hydrogels	Moderate	Low	Efficient for tissue repair, drug delivery, and wound healing due to ionic interactions that enhance biocompatibility and adhesion.	Excellent biocompatibility with biological systems due to ionic interactions. Suitable for wound healing and tissue repair applications, providing good adhesion. They can self-heal and reassemble under certain conditions, making them reusable in some cases.	Sensitive to ionic strength and pH changes in the environment, which can destabilize their performance. Limited long-term stability, especially in dynamic biological environments. May request reinforcement to improve mechanical strength.	[82,83,84,85,86]

**Table 2:** Summary of the chronic wound application of hydrogels.

Hydrogel Material	Additional Bioactive Components	Testing Stage	Experimental Results	Ref.
Collagen hydrogel	Hydroxypropyl methylcellulose (HPMC) and Polyvinyl alcohol (PVA)	In vivo (rats)	The experimental group had a larger healing area than the positive control group on days 14 and 21. In diabetic rats, collagen gel dressing can accelerate and improve the quality of full-thickness wound healing.	[100]
Poly(polyethylene glycol citrate co-N-isopropylacrylamide) (PPCN) hydrogel	Stromal cell-derived factor-1 (SDF-1)	In vitro and in vivo (mice)	DFU wounds treated with PPCN + SDF-1 showed faster healing (24 days), increased granulation tissue development, epithelial maturation, and the most perfused blood vessels.	[108]
Multi-arm thiolated polyethylene glycol (SH-PEG) with silver nitrate (AgNO3) hydrogel (Ag-SH-PEG hydrogel)	Desferrioxamine (DFO)	In vitro and in vivo (mice)	The hydrogel effectively treated diabetic skin lesions with minimal bacterial infection and increased angiogenic activity.	[103]
N-isopropylacrylamide (NIPAM)-based, thermosensitive hydrogel	Bone marrow mesenchymal stem cells (BMSCs)	In vitro and in vivo (mice)	On day 7, the hydrogel-loaded MSC group’s average unhealed area in type II diabetic mice was 24.6 ± 4.21%, much less than the mean of the untreated control group (79.54 ± 5.92%), suggesting that hydrogel stimulated angiogenesis, granulation tissue creation, re-epithelialization, and even hair follicle and sebaceous gland regeneration.	[109]
Gellan gum-PEG-chitosan hydrogel (GGCH-HGs)	Apigenin (APN)	In vivo (mice)	APN-loaded GGCH-HGs had a strong antioxidant impact and a greater wound-healing effect in both diabetic and healthy wound tissues.	[110]
Alginate (Alg) hydrogel	human umbilical cord-derived outgrowth endothelial cells (OECs), substance P and neurotensin	In vitro and in vivo (mice)	The hydrogel accelerated wound closure when compared to alginate gel alone, and the combination of OEC and SP proved to be the most effective.	[101]
Quaternized chitosan (QCS)-oxidized starch (OST) hydrogel	Exosomes	In vivo (mice)	Nucleus pulposus (NP) cell senescence was rejuvenated by QCS-OST/Exos hydrogel, encouraging extracellular matrix (ECM) remodeling and partially restoring the NP and annulus fibrosis structures.	[102]
Hydrogel made of hyperbranched multi-acrylated poly(ethylene glycol) macromers (HP-PEGs) and thiolated hyaluronic acid (HA-SH)	Adipose-derived stem cells (ADSCs)	In vitro and in vivo (subcutaneous implantation and mice)	This adaptable hydrogel system with ADSCs demonstrated an accelerated diabetic wound healing process by reducing inflammation and encouraging angiogenesis and re-epithelialization.	[104]
PVA-Alg hydrogel (H)	Green tea polyphenol nanospheres (TPN)	In vitro and in vivo (mice)	TPN@H promotes wound healing and regulates immune response by controlling the PI3K/AKT signaling pathway.	[105]
Poly(vinyl pyrrolidone) (PVP)/Alginate/Chitosan hydrogel	Silver nanoparticles	In vitro	The results demonstrate that the 10 mM AgNP-based hydrogel has the best antibacterial properties while maintaining non-cytotoxicity, confirming its suitability for use in pressure ulcer therapy cases.	[106]

**Table 3:** Essential features of hydrogel matrices filled with active ingredients that respond to stimuli.

Hydrogel-Modifying Substance	Main Characteristics of Modified Matrices	Refs.
Polyphenols	Adhesion Mechanical strength Structural integrity Strong elasticity Hemostatic properties Self-healing characteristics Antimicrobial properties Antioxidant properties Anti-inflammatory characteristics	[148,149,150]
Chitosan	Exhibition of a moist wound environment Protection against infections Promotion of leukocyte activity for wound exudate disposal Regulation of degradation through deacetylation Reduced scar tissue development	[151,152]
Peptides, polypeptides, proteins, and amino acids	Stimulation of regeneration mechanisms Biocompatibility Antimicrobial properties	[153,154]
Polysaccharides	Biocompatibility Biodegradability Bioactive properties Facilitated angiogenesis for wound healing	[155,156]
Metal oxides	Antimicrobial, antioxidant, and anti-inflammatory properties	[157,158]
Silver nanoparticles	Stability Antimicrobial and anti-inflammatory properties Resilience	[159,160]
Synthetic polymer materials	Mechanical stability Tunable properties Shortened inflammatory stage of the healing process Controlled drug delivery	[161,162]
Antibiotics	Antibacterial properties	[163,164]

**Table 4:** An overview of phage-delivering hydrogels in soft tissue infections and skin damage.

Therapeutic Use	Hydrogel	Target Bacteria	Phages	Testing Level	Findings	Refs.
Treating wounds related to burn injuries	HPMC hydrogel	*K. pneumoniae*	Kpn5	In vivo (mice)	The greatest survival rate in contrast to gentamicin and silver nitrate after 7 days.	[168]
	PVA-SA hydrogel	*S. aureus* *P. aeruginosa* *K. pneumoniae*	MR10 PA5 Kpn5	In vitro and in vivo (mice)	Demonstrated a decrease in inflammation with wound contraction and a significant reduction (>1 log reduction) in resistant burn wound infection.	[167]
Treatment for skin infections	Agarose-HAMA hydrogel	*S. aureus*	Phage K	In vitro	Hyaluronidase release of phage K damages the HAMA layer and inhibits bacterium development.	[165]
	HA-PEG hydrogel	*P. aeruginosa*	PAML-31-1 LPS-5 Luz24	In vitro and in vivo (mice)	Hydrogel-based sustained phage administration provides a useful, well-tolerated alternative for topical treatment while improving phage therapy’s effectiveness.	[169]
	PVA hydrogel	*E. coli*	UFV-AREG1	In vitro	Compared to the PVA control, the PVA-phage inhibition zone was more significant (*p* < 0.05), suggesting that it can be used for the treatment of skin infections.	[170]
Treatment for skin and/or soft tissue infection	PNIPAM-co-ALA hydrogel	*S. aureus*	Phage K	In vitro	At 37 °C, PNIPAM-co-ALA nanogels coupled to phage K demonstrated thermally induced bacterial lysis of S. aureus.	[166]
	Sodium alginate (SA)-Carboxymethyl cellulose (CMC) -Hyaluronic acid (HA) hydrogel	*E. faecium*	EF-M80	In vivo (mice)	It has been shown that the EF-M80 phage maintained its antibacterial qualities, lysing E. faecium efficiently in the host environment. Its ability to promote wound healing was further increased by encapsulating it in a hydrogel delivery method.	[171]
	Alginate hydrogel	*S. aureus*	Genetically modified phage	In vitro and in vivo (rat)	Significantly reduced soft tissue infection (>0.5 log reduction).	[172]
